# TRPC3 Is Dispensable for β-Alanine Triggered Acute Itch

**DOI:** 10.1038/s41598-017-12770-0

**Published:** 2017-10-24

**Authors:** Peter Dong, Changxiong Guo, Shengxiang Huang, Minghong Ma, Qin Liu, Wenqin Luo

**Affiliations:** 1 Department of Neuroscience, Perelman School of Medicine, University of Pennsylvania, Philadelphia, PA 19104 USA; 2 Department of Anesthesiology and the Center for the Study of Itch, Washington University School of Medicine, St. Louis, MO 63110 USA; 3 Wuhan University, Wuhan, Hubei China

## Abstract

The detection of pruritic (itchy) stimuli is mediated by a variety of receptors and channels expressed by primary sensory neurons. The G protein-coupled receptor (GPCR) MRGPRD is selectively expressed by a subset of mouse non-peptidergic nociceptors and functions as the molecular receptor for the itch-inducing chemical β-alanine. However, the channels responsible for generating electrical signals downstream of MRGPRD remain unclear. Here, we found that a member of the canonical TRP channel family, TRPC3, is highly expressed in MRGPRD^+^ non-peptidergic nociceptors, raising the possibility of whether TRPC3 functions as a downstream channel in the MRGPRD signaling pathway. We tested *TrpC3* null mice for β-alanine induced itch, and found that these mice exhibit normal responses to β-alanine. At the cellular level, calcium influx triggered by β-alanine is also unchanged in cultured DRG neurons from *TrpC3* null mice compared to wild type. Together, our results demonstrate that mouse *TrpC3* is dispensable for β-alanine-induced acute itch.

## Introduction

Pruritoception, the sensation tuned for the detection of itchy stimuli, alerts an organism to harmful external threats such as parasites and toxins. Chronic itch accompanies a wide range of pathological conditions such as multiple sclerosis, neuropathy, and shingles, which is often resistant to treatment, and severely impacts patients’ quality of life^1–3^. Thus, understanding the molecular mechanisms underlying itch sensation is of highly relevant to human health.

Transient receptor potential (TRP) channels comprise a superfamily of more than 30 membrane-bound proteins that form nonselective cation channels when assembled into homo- or hetero-tetramers. These TRP channels detect temperature, pH, osmolality, mechanical stimuli, and various endogenous and exogenous ligands, and play prominent functional roles in sensory signaling in mammals^4,5^. A number of these channels, including TRPA1, TRPM8, and TRPV1, are highly expressed by primary sensory neurons and mediate thermal, cold, pain, and chemical sensations^6–8^. Additionally, these channels can also be activated by GPCR-mediated intracellular signaling cascades and initiate neuronal depolarization, particularly in the context of itch sensation. For example, TRPA1 is suggested to function downstream of the GPCR MRGPRA3 for the detection of chloroquine induced itch^9^, and TRPV1 is proposed for the detection of histamine induced itch through the GPCR H1HR^10^.

In our study, we observed specific and high expression of a canonical TRP channel family member, TRPC3, in MRGPRD^+^ non-peptidergic, C fiber nociceptors. TRPC3 has previously been identified as a mediator of light touch in DRG neurons, although its specific expression pattern was unclear^11^. More recent data demonstrated a role for TRPC3 in vestibular functions^12^. In addition to these functions, TRPC3 is involved in store-operated calcium entry in DRG neurons and thus is likely to function downstream of receptors that respond to inflammatory compounds^13^. Indeed, TRPC3 has been shown to be required for the cellular response to IgG immune complex (IgG-IC), a pain-inducing inflammatory compound that binds to the GPCR FcγRI, which in turn is coupled to TRPC3 through the Syk-PLC-IP_3_ pathway^14^. However, the role of TRPC3 in itch has not yet been investigated.

MRGPRD is a G_q_-coupled GPCR that mediates β-alanine-induced itch sensations^15,16^. Unlike most other characterized mouse MRGPR receptors, which are typically expressed by very restricted populations of itch-dedicated C-fiber sensory neurons, MRGPRD is broadly expressed by non-peptidergic C-fibers and comprise approximately 20% of total neurons in dorsal root ganglia (DRG) and trigeminal ganglia (TG). Furthermore, MRGPRD expression marks a unique population of polymodal sensory neurons that detect mechanical, thermal, and chemical stimuli. The downstream signaling mechanisms of MRGPRD remain elusive. Given the high degree of co-expression we found between TRPC3 and MRGPRD, we hypothesized that TRPC3 functions as a downstream transduction channel of MRGPRD to provide depolarizing signal, or to amplify signals.

We tested behavioral responses of *TrpC3* null mice and *ex vivo* physiology of *TrpC3* null sensory neurons. Our studies show that *TrpC3* null mice do not exhibit any significant defects in the detection of the pruritogen β-alanine. Calcium responses of non-peptidergic nociceptors to β-alanine is also unchanged in the absence of TRPC3, indicating that TRPC3 is dispensable in acute MRGPRD sensory signal transduction. Taken together, our results reveal that the deletion of *TrpC3* on its own is not sufficient to significantly impact β-alanine induced itch responses in non-peptidergic DRG neurons.

## Results

### Expression of *TrpC3* in dorsal root ganglion (DRG) neurons

**Figure 1:**
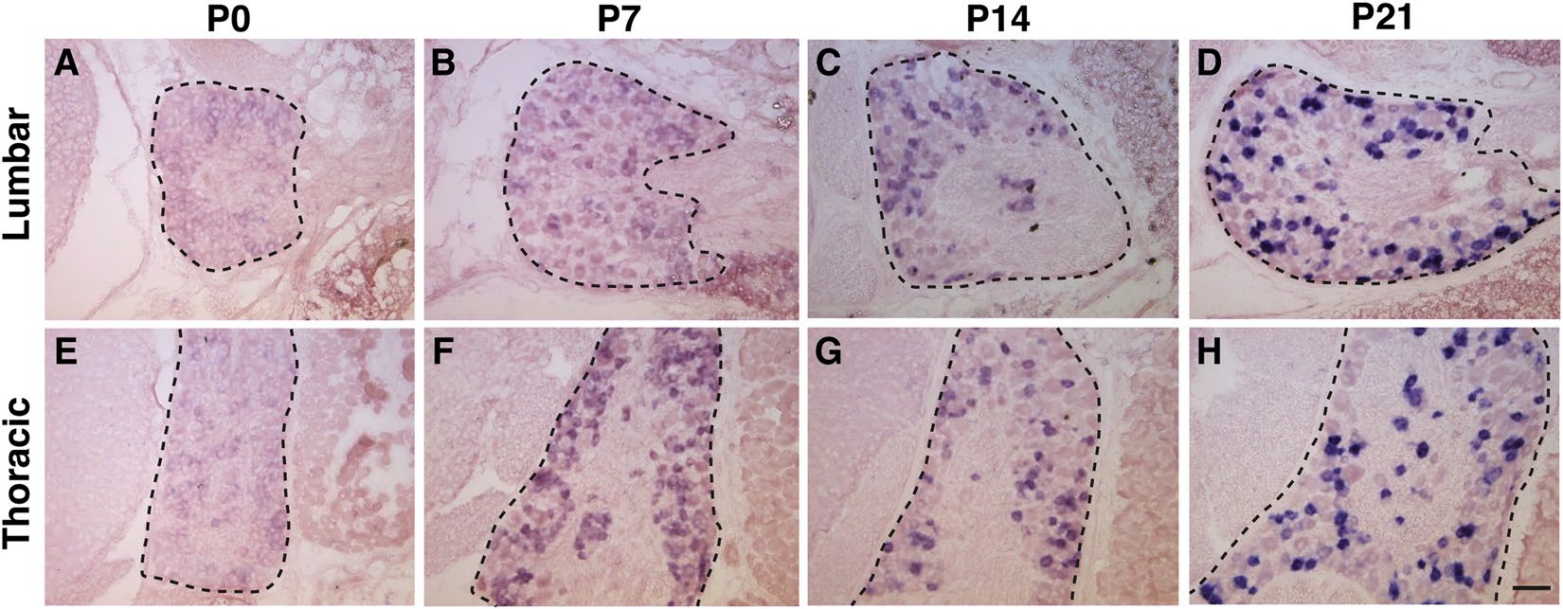
Expression of *TrpC*3 in thoracic and lumbar level DRG neurons. (**A**–**D**) AP colorimetric *in situ* hybridization for *TrpC3* in P0–P21 WT mouse DRG thoracic sections. (**E**–**H**) AP colorimetric *in situ* hybridization for *TrpC3* in P0–P21 WT mouse DRG lumbar sections. In DRGs from both levels, *TrpC3* expression is low at birth and reaches a high level at P21. DRG is outlined by the dashed black line. N = 3 mice for each age. Scale bar = 50 μm.

**Figure 2:**
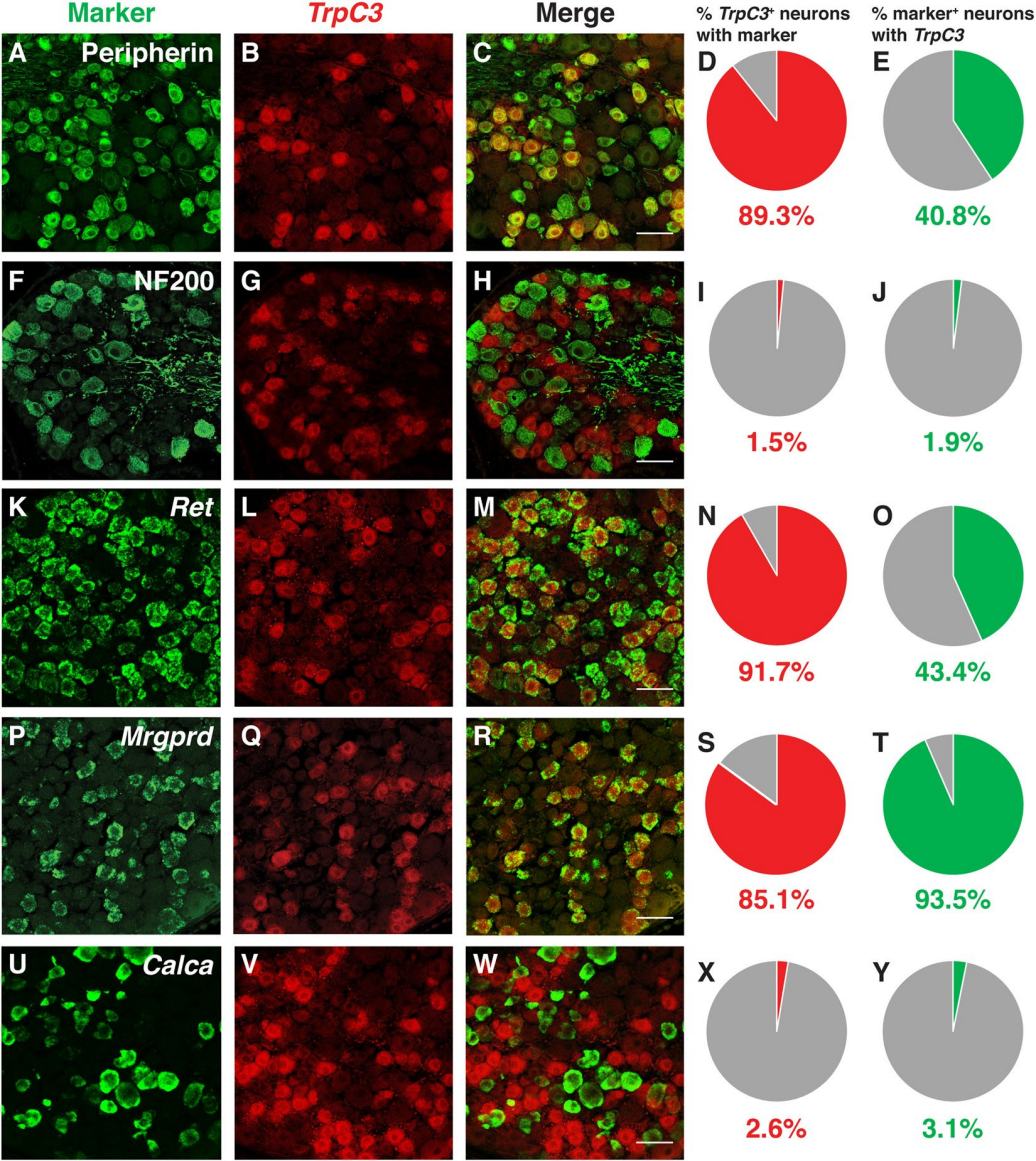
Characterization of *TrpC3* expression in DRG neurons. (**A**–**J**) Fluorescent *in situ* hybridization for *TrpC3* and immunostaining using antibodies against peripherin (**A–E**) and NF200 (**F**–**J**), along with percentage overlap quantification, in P21 WT mouse DRG thoracic sections. (**K–Y**) Double fluorescent *in situ* hybridization for *TrpC3* and *Ret* (**K–O**), *Mrgprd* (**P–T**), and *Calca* (**U–Y**), along with percentage overlap quantification, in P21 WT mouse DRG thoracic sections. *TrpC3* overlaps with the small diameter DRG neuron marker peripherin and the non-peptidergic neuron markers *Ret* and *Mrgprd*, but shows minimal overlap with the large diameter DRG neuron marker NF200 and the peptidergic neuron marker *Calca*. N = 3 mice, Scale bar = 50 μm.

To thoroughly characterize the expression pattern of *TrpC3* in DRG neurons, we performed *in situ* hybridization (ISH) on thoracic and lumbar level DRG sections at four time points spaced a week apart from P0 to P21. We found that *TrpC3* is expressed at high levels in both thoracic and lumbar level DRGs at the beginning of adulthood (P21), but its expression is barely detectable at P0, suggesting that expression of *TrpC3* steadily increases during postnatal development (Fig. 1A–H). Since *TrpC3* expression is comparable between thoracic and lumbar levels, we then used thoracic level DRGs to perform fluorescent *in situ* hybridization (FISH) for *TrpC3*, and FISH or immunostaining for known DRG neuron markers and/or other genes implicated in sensory signaling. We quantified the percentage of *TrpC3* ^+^ neurons that co-express the selected marker, as well as the percentage of marker^+^ neurons that co-express *TrpC3*. We found that the vast majority of *TrpC3* ^+^ neurons are positive for peripherin (89.3%), a marker for small-diameter neurons (Fig. 2A–E), while almost no *TrpC3* ^+^ neurons are positive for NF200 (1.5%), a marker for large-diameter neurons such as Aβ mechanoreceptors and proprioceptors (Fig. 2F–J). In addition, almost all *TrpC3* ^+^ neurons co-express markers for non-peptidergic neurons such as *Ret* (91.7%) and *Mrgprd* (85.1%) (Fig. 2K–T). On the other hand, *TrpC3* ^+^ neurons rarely express *Calca* (2.6%), the gene that codes for CGRP, a neuropeptide found largely in peptidergic neurons (Fig. 2U–Y). Interestingly, almost all *Mrgprd* ^+^ neurons (93.5%) also express *TrpC3* (Fig. 2P–T).

**Figure 3:**
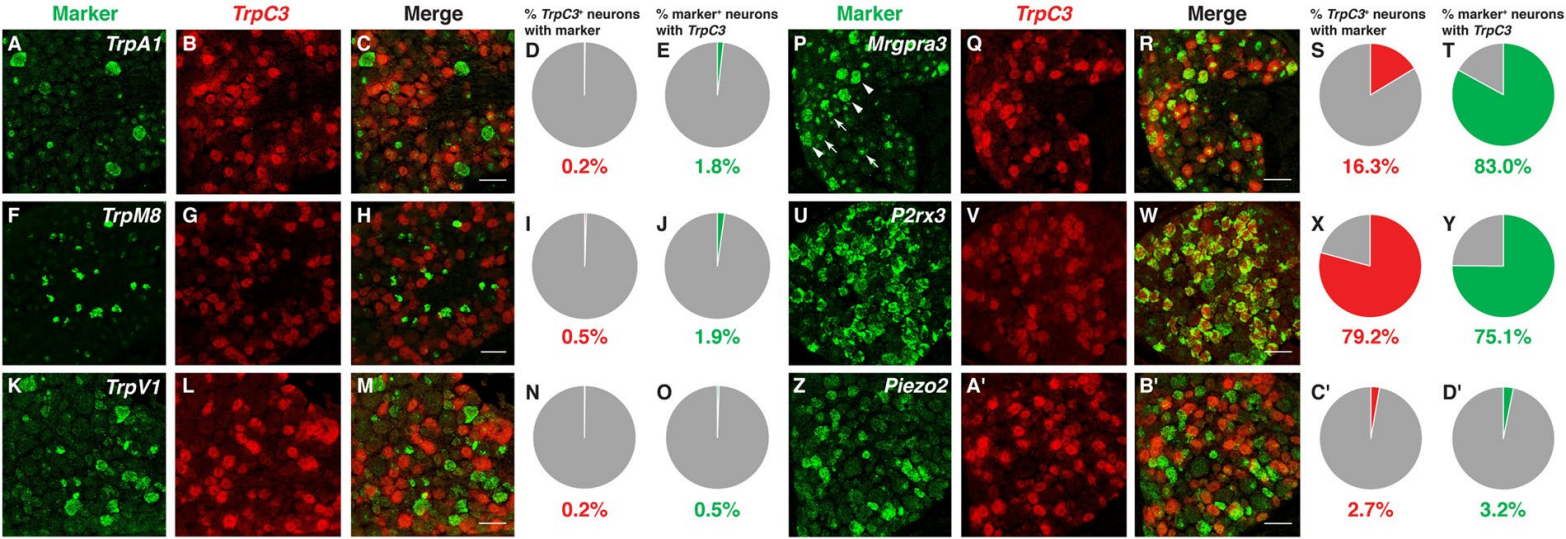
Expression of *TrpC3* in DRG neurons with regard to other channels and receptors involved in somatosensation. (**A–D**’) Double fluorescent *in situ* hybridization for *TrpC3* and *TrpA1* (**A**–**E**), *TrpM8* (**F**–**J**), *TrpV1* (**K**–**O**), *MrgprA3* (**P**–**T**), *P2rx3* (**U**–**Y**), and *Piezo2* (**Z**–**D’**), along with percentage overlap quantification, in P21 WT mouse DRG thoracic sections. Few *TrpC3* ^+^ DRG neurons express other TRP channel genes, as well as *MrgprA3* and *Piezo2* (although most *MrgprA3* neurons express *TrpC3*). However, *TrpC3* overlaps highly with *P2rx3*, a receptor for ATP. In some FISH, there is green nuclear background (white arrows), which can be differentiated from real cytosolic signals (white arrowheads). N = 3 mice. Scale bar = 50 μm.

We then performed double fluorescent ISH using RNA probes against *TrpC3* and genes coding for other known channels and receptors that mediate sensory signaling in DRG neurons. We first looked at the expression of *TrpC3* in conjunction with other TRP channels expressed at high levels in DRG neurons. *TrpC3* showed minimal overlap (<1%) with *TrpA1*, *TrpM8*, or *TrpV1* (Fig. 3A–O). A previous publication showed that MRGPRA3, another member of the MRGPR family of GPCRs and the receptor for chloroquine, is coupled to TRPA1 for signaling^9^. Here, we found that most neurons which express *MrgprA3* (83.0%) also express *TrpC3* (Fig. 3P–T), raising the possibility that TRPC3 could play a previously undescribed role in this population of neurons as well. The calcium permeable channel P2X3, encoded by the *P2rx3* gene, is an ATP receptor^17,18^ expressed largely in non-peptidergic nociceptors. We found that the majority of *TrpC3* ^+^ neurons (79.2%) also express *P2rx3* (Fig. 3U–Y). Lastly, the channel PIEZO2 has recently been implicated in the detection of mechanical force in DRG neurons as well as Merkel cells in the skin^19,20^. Little overlap is detected between *TrpC3* and *Piezo2* (<4%), suggesting that either PIEZO2 is not the channel mediating noxious mechanical force in this population of neurons, or these neurons express a very low level of *Piezo2*, which is below the detection threshold of FISH (Fig. 3Z–D’). Taken together, our results indicate that *TrpC3* is highly and specifically expressed in MRGPRD^+^ and MRGPRA3^+^ non-peptidergic nociceptors.

### Characterization of *TrpC3* expression in somatosensory neurons of *TrpC3* null mice

**Figure 4:**
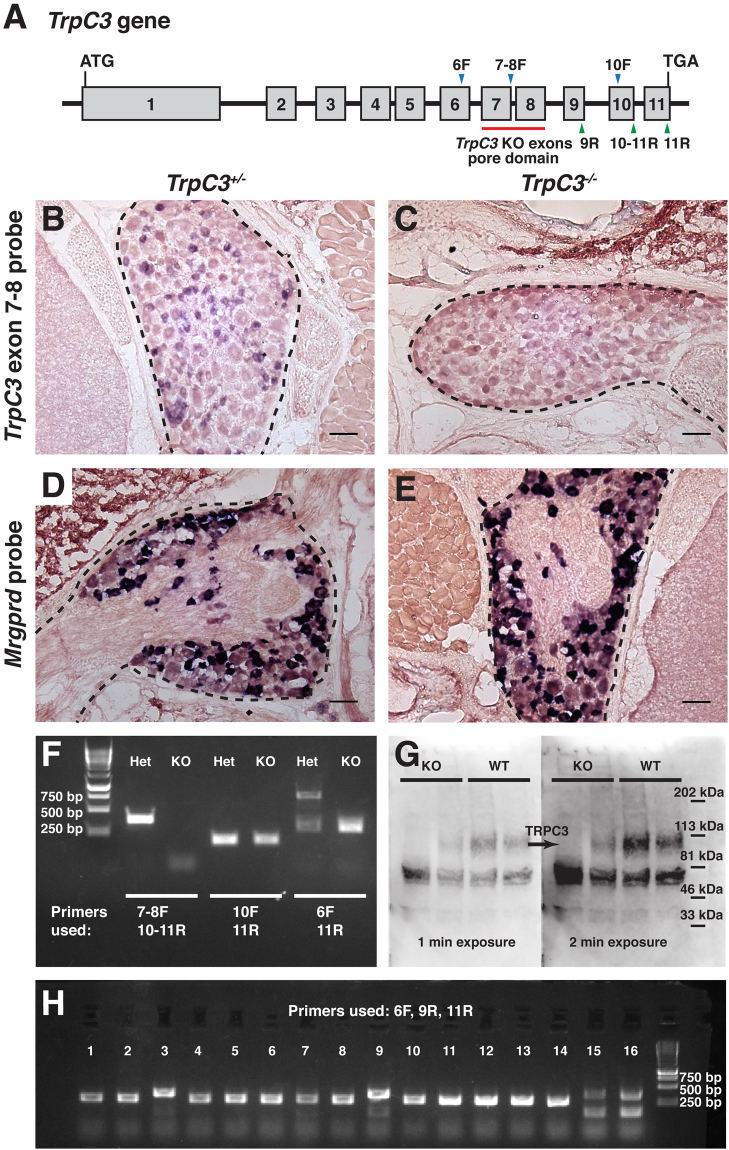
Characterization of the *TrpC3* knockout mice. (**A**) Diagram of the *TrpC3* gene, depicting the 11 exons, of which 7–8 are excised in the KO. Also shown are the primers used in RT-PCR and colony PCR experiments, which include forward primers recognizing exon 6, the junction of exons 7–8, and exon 10 (6 F, 7–8 F, 10 F) and reverse primers recognizing exons 9, 11, and the junction of exons 10–11 (9 R, 11 R, 10–11 R). (**B** and **C**) AP colorimetric *in situ* hybridization with a *TrpC3* probe that recognizes exons 7 and 8 of *TrpC3* transcript in *TrpC3* heterozygote and KO mouse DRG thoracic sections. Probe signal is seen in the *TrpC3* heterozygote DRG but not in the KO. (**D** and **E**) AP colorimetric *in situ* hybridization with an *Mrgprd* probe in *TrpC3* heterozygote and KO mouse DRG thoracic sections. No obvious difference in signal is seen between the *TrpC3* heterozygote and KO. For panel 4B–E, DRG is outlined by the dashed black line. Scale bars = 50 μm. (**F**) RT-PCR performed on RNA acutely isolated from *TrpC3* heterozygote and KO DRG neurons. cDNA was amplified with primers specific for (from left to right): forward primer at the junction of exons 7–8 and reverse primer at the junction of exons 10–11 (WT: 387 bp, KO: no band), forward and reverse primers specific for exons 10 and 11 (WT: 194 bp, KO: 194 bp), and forward primer a exons 6 and reverse primer at the junction of exons 10–11 (WT: 783 bp, KO: 377 bp). (**G**) Western blotting performed on cell lysate isolated from WT and *TrpC3* KO DRG neurons from two mice per genotype, taken after 1 and 2 minutes of exposure time. TRPC3 protein was detected in WT DRG neuron extract using a TRPC3 N-terminus antibody, with an expected size of 95.7 kDa. (**H**) Colony PCR performed on bacteria transformed with plasmids containing exon 6–11 RT-PCR products from a *TrpC3* KO mouse. The colony PCR was conducted using primers specific for exon 6 (forward primer), exon 9 (reverse primer 1), and exon 11 (reverse primer 2). out of 16 colonies (25%) contain two bands: the smaller band (151 bp) for exons 6 and 9, and the larger band (377 bp) for exons 6, 9, 10, and 11. In the remaining 12 colonies (75%), one band was observed: a 293 bp PCR product for exons 6, 10, and 11. PCR products were sequenced to confirm their components. N = 3 mice for all experiments.

Given the high degree of overlap between *TrpC3* and *Mrgprd*, we decided to examine the functional relationship between these two signaling proteins. From Dr. Barbara Miller at Penn State University, we obtained *TrpC3* knockout (KO) mice, in which exons 7 and 8 coding for the pore domain of TRPC3, are excised (Fig. 4A)^21^. We generated an RNA probe for exons 7 and 8 of *TrpC3* and performed AP colorimetric ISH on P21 thoracic DRG sections from *TrpC3* KO and heterozygous animals. We observed that the *TrpC3* exon 7 and 8 signal was completely lost in the KO DRGs (Fig. 4B,C), but the expression of *Mrgprd* was unaffected (Fig. 4D,E), indicating that exons 7 and 8 of *TrpC3* are ablated in DRG neurons as expected and that this manipulation does not cause cell death of non-peptidergic nociceptors. In a complementary experiment, we conducted RT-PCR using DRG RNA extract of *TrpC3* KO mice. We found that exons 7–8 but not 10–11 of *TrpC3* transcript is lost in these mutant mice (Fig. 4F). To determine that truncated *TrpC3* mRNAs could not generate functional proteins, we subcloned *TrpC3* RT-PCR products (exons 6–11) from *TrpC3* null mice and sequenced multiple clones. Interestingly, the majority (~75%) of *TrpC3* transcripts lack exons 7–9 while ~25% of transcripts lack exons 7 and 8 (Fig. 4H). Premature stop codons are found shortly after exon 6 in both forms of transcripts (data not shown). Since exons 7 and 8 encode the channel pore domain, no functional TRPC3 protein could be made from these truncated *TrpC3* transcript in this mutant mouse line. Finally, we examined the presence of TRPC3 protein in DRG neurons by Western blot using an N-terminus TRPC3 antibody (a gift from Dr. Craig Montell at UC Santa Barbara). Consistent with our mRNA transcript analysis, we found that a full-length band is present in WT lysates but absent in the KO DRG cell lysates (Fig. 4G). Nonspecific bands appear at other molecular weights, but signal appears to be similar across WT and KO lysates. Collectively, our results suggest that the truncated TRPC3 protein lacking exons 7–8 or 7–9 is either untranslated or degraded in the DRG neurons and that the *TrpC3* KO mice we obtained are true *TrpC3* null mice.

### *TrpC3* is not required for the development of non-peptidergic nociceptors

**Figure 5:**
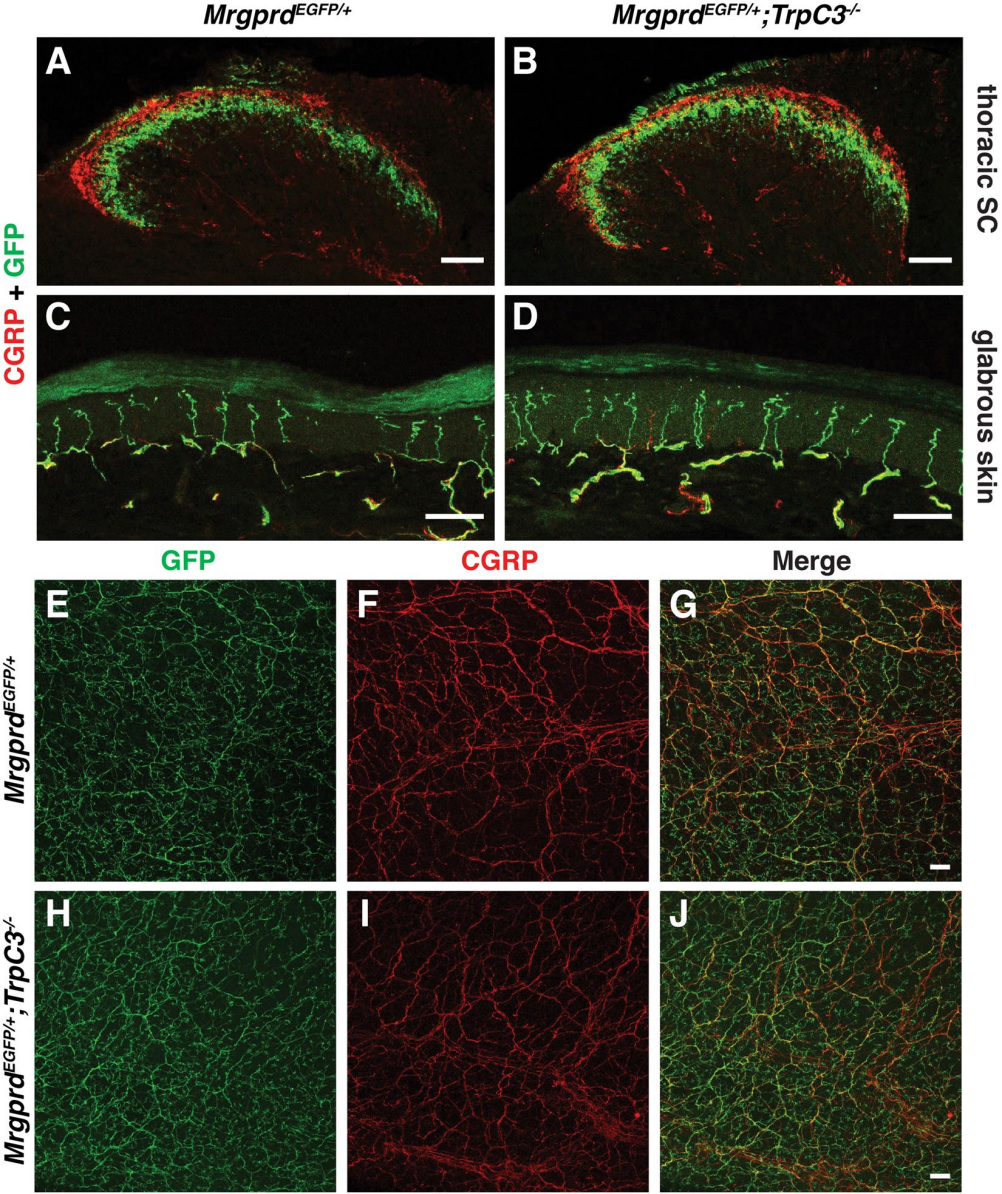
Gross anatomy of non-peptidergic nociceptors in the spinal cord and skin is normal in *TrpC3* null mice. (**A** and **B**) Immunostaining in P21 *Mrgprd* ^*EGFP/*+^ and *Mrgprd* ^*EGFP/*+^; *TrpC3* ^−/−^ mouse thoracic spinal cord sections using antibodies against CGRP and GFP. (**C** and **D**) Immunostaining in P21 *Mrgprd* ^*EGFP/*+^ and *Mrgprd* ^*EGFP/*+^;*TrpC3* ^*−/−*^ mouse glabrous skin cross sections using antibodies against CGRP and GFP. (**E**–**J**) Immunostaining in P21 *Mrgprd* ^*EGFP/*+^ and *Mrgprd* ^*EGFP/*+^;*TrpC3* ^*−/−*^ mouse whole mount glabrous skin using antibodies against CGRP and GFP. No obvious differences are seen in spinal cord and peripheral nerve endings between WT and *TrpC3* KO backgrounds. N = 3 mice for each genotype. Scale bars = 50 μm.

While many TRP family members have roles in detecting sensory stimuli, several canonical TRP channels are also required for proper nervous system development in processes such as axon growth cone guidance and neuronal survival^22,23^. Whether TRPC3 has an effect on the development of DRG neurons has not been determined. To examine the role of TRPC3 in DRG neuron development and somatosensation, we examined gross anatomy of non-peptidergic nociceptors in *TrpC3* KO mice. We took advantage of the almost complete overlap between *Mrgprd* and *TrpC3* expression observed in the ISH data, and examined the morphologies of *Mrgprd* ^+^ neurons using a mouse line in which an enhanced green fluorescent protein (EGFP) reporter is expressed from the *Mrgprd* locus (*Mrgprd* ^*EGFP*^)^24^. We generated *Mrgprd* ^*EGFP*/+^ *; TrpC3* ^*−/−*^ null mice and littermate *Mrgprd* ^*EGFP/*+^ controls and performed fluorescent immunostaining using an anti-GFP antibody to label central and peripheral axons of *Mrgprd* ^+^ DRG neurons, as well as an anti-CGRP antibody to label those of peptidergic nociceptors in the spinal cord and skin, which served as a negative control. As reported previously^24^, we observed that CGRP^+^ peptidergic nociceptor axons innervate lamina I of the dorsal horn while the MRGPRD^+^ non-peptidergic nociceptor endings innervate lamina II of the dorsal horn. In addition, this central projection pattern is very similar between *TrpC3* KO and control animals in terms of shape or size (Fig. 5A,B). In the skin, non-peptidergic fibers terminate in the stratum granulosum of the epidermis, where they wind around keratinocytes and form a distinctive zigzag shape^24^. These axon endings lie superficial to the peptidergic axon endings, which display straight trajectories into the stratum spinosum. We found that termination patterns in the skin also showed no obvious differences in morphology between the *TrpC3* KO and control animals using cross sections of glabrous (nonhairy) skin (Fig. 5C,D). Furthermore, when we examined skin innervation patterns over the entire epidermal surface using whole mount skin staining of glabrous skin from the hindpaw, our results are consistent with those above (Fig. 5E–J). These results, along with our ISH data showing that full expression of *TrpC3* does not occur until P21 (Fig. 1A–H), and previously published RT-PCR results showing a sustained high level of *TrpC3* expression in adult mouse DRGs^25^, suggest that TRPC3 likely functions in mature non-peptidergic nociceptors but is not required for the normal development of this population of neurons.

### The role of TRPC3 in β-alanine induced itch

**Figure 6:**
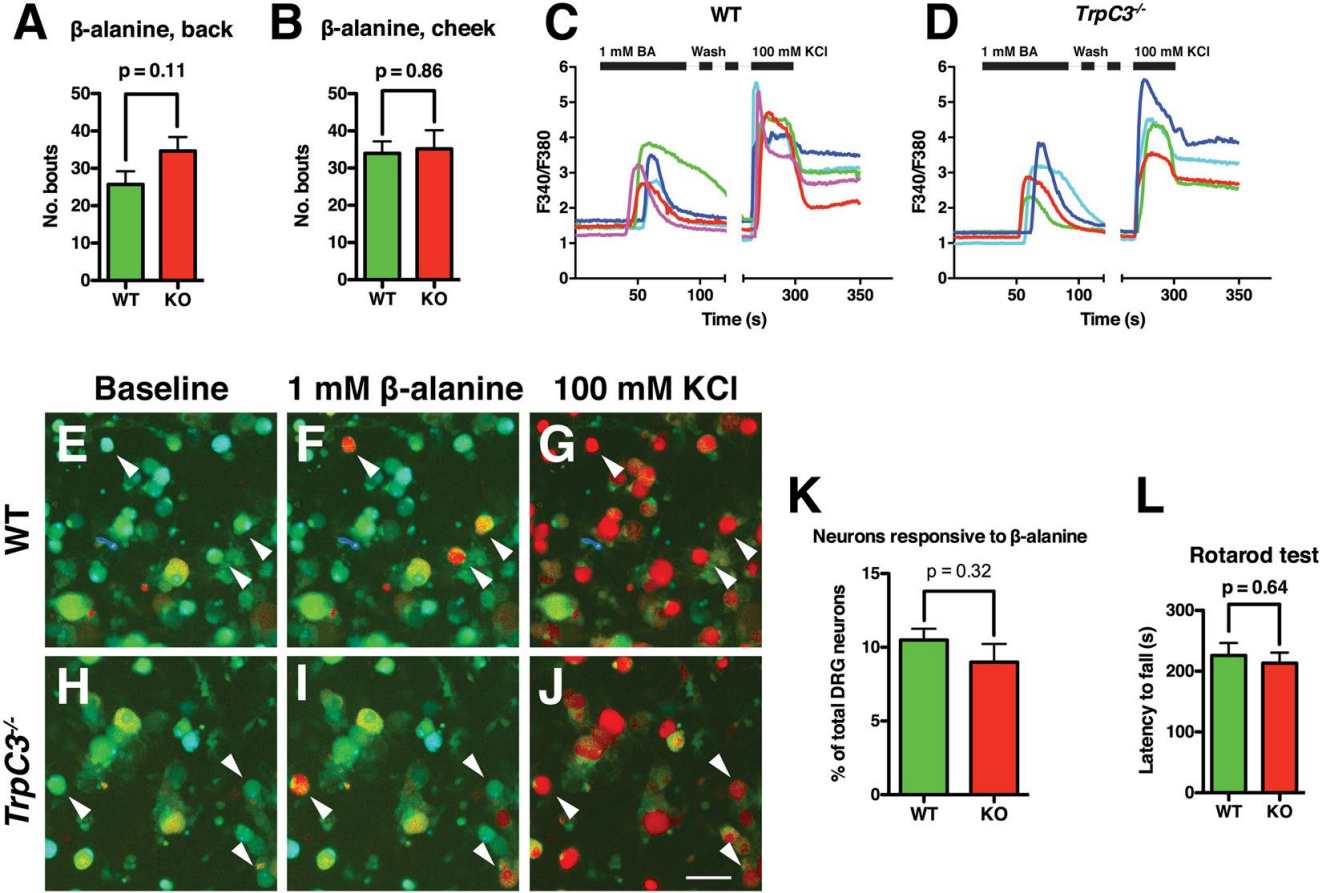
TRPC3 is not required for MRGPRD-evoked behavior or signaling at a cellular level. (**A,B**) Adult (>6 weeks old) *TrpC3* KO and littermate WT control mice were scored for scratching behavior after β-alanine injection into the cheek and back. N = at least 7 mice per genotype for β-alanine back injection, at least 5 mice per genotype for β-alanine cheek injection. (**C**,**D**) Representative traces of calcium activity from littermate control and *TrpC3* ^*−/−*^ null cultured DRG neurons. DRG neurons of both genotypes show normal responses to β-alanine, while activity in response to KCl demonstrates that the neurons are healthy and viable. (**E–J**) Representative images of DRG neurons from control (**E–G**) and *TrpC3* ^*−/−*^ null (**H–J**) mice, with arrowheads indicating neurons that show responsiveness to both 1 mM β-alanine and 30 mM KCl. Percentages were pooled from results collected from 1,481 control and 990 *TrpC3* KO DRG neurons from 6 animals per genotype. Scale bar = 50 μm. (**K**) Bar graph comparing the percentage of all DRG neurons that showed a positive response to β-alanine between littermate control and *TrpC3* ^*−/−*^ null cultured DRG neurons. No significant difference was seen (9.9 ± 0.8% in control DRG neurons vs. 9.3 ± 1.2% in *TrpC3* KO DRG neurons; student *t* test). (**K**) Adult (>6 weeks old) *TrpC3* KO and littermate WT control mice were scored for latency to fall off rotarod apparatus. N = 9 mice per genotype.

TRPC3 is expressed in mature somatosensory neurons, but its function in itch behavior remains to be determined. MRGPRD^+^ non-peptidergic neurons are required to mediate β-alanine induced itch, and mice lacking MRGPRD no longer display an itch response to β-alanine^16^. We utilized *TrpC3* KO (in C57 background) and littermate control mice to determine whether TRPC3 plays a role β-alanine induced itch. *TrpC3* KO mice are fertile, healthy, live through adulthood, and show no differences in general behaviors compared to wild type (WT) mice (data not shown). To avoid potential confounds in our behavioral experiments, we conducted a rotarod test to verify that *TrpC3* KO mice show no deficits in motor behavior. No significant difference was seen in latency to fall times between the two groups, indicating that *TrpC3* KO mice exhibit normal motor and learning behavior (Fig. 6L). While a previous study found deficits in motor coordination in *TrpC3* KO mice^26^, some recent data support our rotarod results and suggest that *TrpC3* might play more of a role in vestibular function^12^.We then performed itch assays in which 50 mM β-alanine was injected into either the cheek or back of *TrpC3* KO or control mice, and found that the scratch bout number was not significantly different between the two genotypes regardless of injection location (Fig. 6A,B). Thus, TRPC3 does not seem to be required for β-alanine evoked itch behavior. In addition, consistent with previously published data^11^, we found no differences in the ability of KO and WT animals to detect thermal and mechanical noxious stimuli using hot/cold plate, Hargreaves, von Frey, and pinprick tests, as well as innocuous touch using the dynamic paintbrush test (data not shown). As a whole, these data support the idea that TRPC3 alone is not required for the acute sensation of temperature or mechanical force^11^.

While TRPC3 does not seem to play a role in the somatosensory behaviors we tested, we wondered if a deficit in sensory signaling could be detected at a cellular level. To determine the functional role TRPC3 plays in non-peptidergic nociceptors and whether TRPC3 functions downstream of MRGPRD signaling, we used calcium imaging to visualize neuronal activity in cultured DRG neurons. We dissociated DRG neurons from *TrpC3* ^*−/−*^ null and control mice, and stimulated the cells with 1 mM β-alanine to activate MRGPRD or with 100 mM KCl as a positive control to verify that the cells were functional and healthy. We found that DRG neurons from *TrpC3* KO and control mice were equally responsive to β-alanine (Fig. 6C–J), and that there was no significant difference in percentage of total DRG neurons responsive to β-alanine (9.9 ± 0.8% in *TrpC3* WT cells vs. 9.3 ± 1.2% in *TrpC3* KO cells; Fig. 6K). This result suggests that TRPC3 is not required for MRGPRD signaling in non-peptidergic neurons.

### *TrpC3* and *TrpC6* are co-expressed in DRG neurons

Since TRPC3 is not necessary for somatosensation in DRG neurons as far as we examined, we wondered if other TRP channels are co-expressed or interact with TRPC3 and thus may compensate for the loss of TRPC3. TRPC6 is another member of the canonical family of TRP channels, and belongs to a subfamily comprising TRPC3, TRPC6, and TRPC7 based on amino acid sequence similarity^27^. TRPC3 and TRPC6 have been shown to physically interact *in vitro*, and evidence suggests that they may form heterotetramers with each other *in vivo* ^28,29^. Furthermore, *TrpC3*/*TrpC6* double KO mice show significantly reduced responses to mechanical force when measured via the von Frey and cotton bud tests^11^. However, direct evidence showing that TRPC3 and TRPC6 are expressed in the same DRG neurons is still lacking.

**Figure 7:**
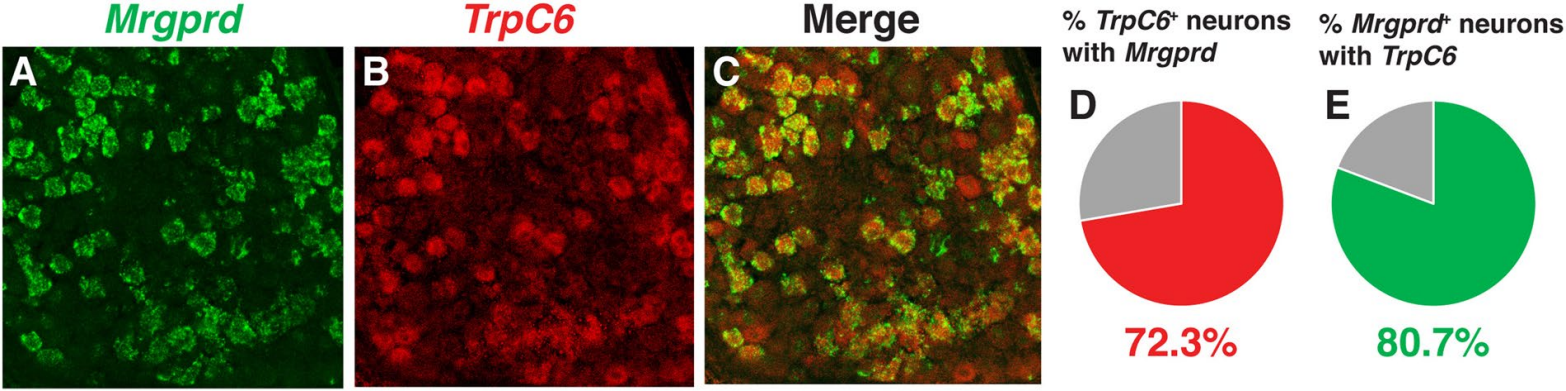
Expression of *TrpC6* in DRG neurons compared to *TrpC3*. (**A**–**E**) Double fluorescent *in situ* hybridization for *Mrgprd* and *TrpC6*, along with percentage overlap quantification, in P21 WT mouse DRG thoracic sections.

We performed double fluorescent ISH with RNA probes against *Mrgprd* and *TrpC6* transcript, and found that *TrpC6* and *Mrgprd* show greater than 70% overlap with each other (Fig. 7A–E). While this overlap is less than that observed between *TrpC3* and *Mrgprd*, this could be explained by the generally weaker signal of *TrpC6* as compared to that of *TrpC3*. Although we were unable to directly show the overlap between *TrpC3* and *TrpC6* due to a sensitivity limitation of FISH, the common high degree of overlap of *TrpC6* and *TrpC3* with *Mrgprd* suggests that overlap between *TrpC3* and *TrpC6* is high as well.

## Discussion

MRGPRD^+^ non-peptidergic nociceptors are polymodal neurons in that they respond to a diverse range of thermal, mechanical, and chemical noxious stimuli, as well as itch inducing compounds. While MRGPRD has been identified as the itch receptor for several chemicals including β-alanine^15^, no candidates for the channel downstream of this GPCR have emerged. Our results (Figs 2P–T, 3 and 7) demonstrate that the TRPC family member TRPC3 is found almost exclusively in MRGPRD^+^ and MRGPRA3^+^ non-peptidergic nociceptors. Though TRPA1 is suggested as a downstream channel for MRGPRA3, we did not find obvious overlap between *TrpC3* and *TrpA1*. This discrepancy could be due to the sensitivity of different methods (calcium imaging versus double fluorescent ISH) or different materials (cultured DRG neurons versus thoracic DRG sections).

It has been previously shown that *TrpC3* KO animals display no deficits in mechanosensation or thermosensation^11^. Our results (data not shown) are consistent with this previous publication. Given the high expression of *TrpC3* in MRGPRD^+^ non-peptidergic nociceptors, in this study, we focused on determining functions of TRPC3 in β-alanine induced itch. We found that TRPC3 is not required for this acute itch sensation as well (Fig. 6A,B). In parallel experiments, when we investigated the function of TRPC3 in MRGRPD signaling at the cellular level using calcium imaging, we found that there was also no difference between cultured DRG neurons from *TrpC3* KO and control mice (Fig. 6C–K). Together, these results demonstrate that TRPC3 is dispensable for MRGPRD signaling at the cellular and behavioral level. We speculate that compensatory mechanisms may exist in this population of neurons, such as TRPC6 (Fig. 7), allowing for the maintenance of normal pain and itch responses even when *TrpC3* is ablated. A previous study utilizing double *TrpC3* and *TrpC6* double KO mice found that double null mice displayed no deficit in the Randall-Selitto test and Hargreaves’ test, but showed significantly reduced response to the von Frey assay and light cotton bud stimulation^11^. Such a deficit suggests that TRPC3 and TRPC6 together may play a role in the sensation of mechanical force. Interestingly, at our hand, *TrpC3* mice also showed a trend toward deficit of light mechanical force (data not shown). Thus, further studies are needed to determine if TRPC3 and TRPC6 function together for the sensation of itch. Intriguingly, ﻿some﻿ recently published work suggested that TRPA1 and TRPV1 may be dispensable for chloroquine- and histamine-evoked cellular responses^30,31^.

There are a few other issues that may also confound the experimental outcomes. The *TrpC3* mutant mice used in the current study and previous publications are straight null mice. Since *TrpC3* is also expressed in the spinal cord and the brain, it is also possible that its function in DRG neurons and somatosensation is somehow masked by deficits in other tissues and cells. Thus, an acute DRG neuron specific ablation of *TrpC3* would be better to address its function. Moreover, recent large-scale analyses of DRG neurons have uncovered a vast array of receptors and channels expressed in non-peptidergic nociceptors^32,33^. TRPC3 may be one of these and other yet uncharacterized detectors of somatosensory stimuli in non-peptidergic nociceptors. Finally, it is possible that though TRPC3 is not required for acute pain and itch sensation, it may play a role in the manifestation of chronic pain or itch phenotypes. Indeed, recent work has demonstrated that TRPC3 lies upstream of the neuronal Fcγ receptor for serum IgG immune complex^14^, suggesting that TRPC3 functions in hyperalgesia. TRPC3 may be downstream of other channels and/or receptors that are active only during pathological conditions.

## Conclusions

We found that *TrpC3* transcript is highly expressed in non-peptidergic nociceptors and shows a high degree of overlap with the itch receptor *Mrgprd* and *MrgA3*, but not with other TRP channel coding genes such as *TrpA1*, *TrpM8*, and *TrpV1*. Despite this specific pattern of expression, TRPC3 is dispensable for β-alanine induced cellular signaling and acute itch behavior via MRGPRD.

## Materials and Methods

### Mouse strains

Mice used at the University of Pennsylvania were raised and housed in a barrier facility in Hill Pavilion, with the exception of mice used for behavior experiments, which were housed in a non-barrier facility in the Smilow Translational Research Center. Mice used at the Washington University School of Medicine were bred and housed in a barrier facility at CSRB-NTA. All procedures were conducted in accordance with animal protocols approved by the Institutional Animal Care and Use Committee (IACUC) of the University of Pennsylvania, the Animal Studies Committee of the Washington University School of Medicine, and National Institutes of Health guidelines. *TrpC3* ^+/−^ mice were provided by Dr. Barbara Miller at Penn State University^21^. *Mrgprd* ^*EGFP/*+^ mice were originally generated by Dr. Mark Zylka at the University of North Carolina^24^. All mice were backcrossed to C57Bl/6 mice spanning several generations and were maintained in a C57Bl/6 background. For all molecular biology, histological, and calcium imaging experiments, at least three animals per genotype were examined and the exact numbers are indicated in the figure legend. For behavior assays, at least five mice per genotype group were used for each assay and the exact numbers are indicated in the figure legend.

### *In situ* hybridization

DIG- and FITC-labeled RNA probes were synthesized using DIG and FITC RNA labeling kits (Roche 11277073910 and 11685619910) as described previously^34^. *In situ* hybridization was performed as described previously^34^. Cervical, thoracic, and lumbar level spinal columns were dissected from euthanized wild type P21 mice and rapidly frozen in OCT (Fisher 6506), then sectioned into 20 μm cryosections. All steps prior to 0.2X SSC wash were carried out under RNase free conditions. 20 μM DRG and spinal cord cryosections were immersion-fixed in freshly made 4% PFA in PBS for 20 minutes at room temperature. Slides were then washed in fresh-DEPC PBS (1:1000 DEPC (Sigma D5758) in 1xPBS immediately before use), followed by wash in DEPC-pretreated PBS (1:1000 DEPC in 1xPBS overnight (O/N), followed by autoclaving) and antigen retrieval. For antigen retrieval, which increases probe signal, citric acid buffer (10 mM citric acid, 0.05% Tween-20, pH 6.0) was boiled in a microwave, and DEPC (1:1000) was added to freshly boiled solution. Slides were immersed in solution in a 95 °C water bath for 20 minutes, and then allowed to cool at room temperature for 30 minutes. Sections were then washed in DEPC-pretreated 1xPBS (1 × 5 minutes), incubated in proteinase K (25 μg/mL in DEPC-pretreated H_2_0) for five minutes, followed by washes in fresh-DEPC PBS (1 × 5 minutes) and DEPC-pretreated PBS (1 × 5 minutes). Sections were then acetylated at room temperature for ten minutes in freshly made acetylation solution (0.1 M TEA, 0.25% acetic anhydride in DEPC-pretreated H_2_0). Slides were then prehybridized in hybridization buffer (50% formamide, 5X SSC, 0.3 mg/mL yeast tRNA, 100 μg/mL heparin, 1X Denhardt’s solution, 0.1% Tween-20, 0.1% CHAPS, 5 mM EDTA in RNase-free H_2_0) at 62 °C in a humidified chamber for 30 minutes. Following pre-hybridization, excess hybridization buffer was removed from slides and 2 ng/μL of riboprobe(s) diluted in hybridization buffer was placed on the slide. Slides were incubated O/N under Parafilm coverslips at 62 °C. Slides were then washed in 0.2X SSC at 68 °C (1 × 15 minutes, 2 × 30 minutes).

For colorimetric reaction, slides were blocked in PBT (PBS, 0.1% Triton X-100) and 20% lamb serum at room temperature for one hour. Sections were then incubated with AP-conjugated anti-DIG antibody (1:1000) in blocking buffer O/N at 4 °C. Slides were washed in PBT (3X 10 minutes) and incubated O/N in darkness in alkaline phosphatase buffer (100 mM Tris pH 9.5, 50 mM MgCl_2_, 100 mM NaCl, 0.1% Tween-20, 5 mM levamisole, 0.34 mg/mL 4-Nitro blue tetrazolium (NBT, Roche 11383213001), 0.17 mg/mL 5-bromo-4-chloro-3-indolyl-phosphate (BCIP, Roche 1138221001)). Following colorimetric reaction, slides were rinsed repeatedly in PBS and then fixed for 20 minutes in 4% PFA in PBS at room temperature. Slides were then repeatedly rinsed in ddH_2_0, dried at 37 °C for 1 hour, dehydrated in xylenes (3 × 2 minutes), and coverslipped with Permount (Fisher SP15).

For double fluorescent ISH, slides were blocked for one hour at room temperature with 0.5% Blocking Reagent in PBS. Sections were incubated in anti-FITC-POD (Roche 11426346910, 1:100 in 0.5% Blocking Reagent) O/N at 4 °C. Slides were then washed in PBT (3X 10 minutes) and incubated in 0.1% BSA in PBS for 15 minutes. FITC RNA probes were then developed using the TSA Plus system (Perkin Elmer NEL741001KT) by diluting fluorescein tyramide into 1X amplification buffer (1:100) and incubating slides in working solution for 10–15 minutes, followed by washes in PBS (3X 10 minutes). Slides were then blocked in PBT containing 20% lamb serum for one hour at room temperature, and incubated O/N at 4 °C with AP-conjugated anti-DIG antibody (Roche 11093274910, 1:500 in PBT and 20% lamb serum). Slides were washed in TNT (100 mM Tris–HCl, 150 mM NaCl, 0.05% Tween-20, pH 7.5) (3X 10 minutes), then in detection buffer (100 mM Tris–HCl, 100 mM NaCl, 10 mM MgCl_2_, pH 8.0) (2X 10 minutes). DIG-labeled probes were then developed using the HNPP/FastRed TR system (Roche 11758888001). Sections were incubated in detection solution (10 μL HNPP stock solution, 10 μL of 25 mg/mL FastRed per 1 mL of detection buffer, filtered through a 0.2 μM nylon filter) (1X 90 minutes). Slides were then rinsed in PBS and mounted with Fluormount (Fisher OB100 01).

For FISH combined with immunofluorescence, normal hybridization procedure was followed, using DIG-labeled probe. After 0.2X SSC washes, sections were blocked for one hour in PBT containing 20% lamb serum. Sections were then incubated with AP-conjugated anti-DIG (1:500) and primary antibody (Aves NUN chicken anti-NF200, 1:500 and Fisher AB1530 rabbit anti-peripherin, 1:2000) at 4 °C O/N in 20% lamb serum blocking solution. Slides were washed in PBT (3X 10 minutes), then incubated in species appropriate Alexa 488 conjugated secondary antibody (1:500 in 5% lamb serum in PBT) for one hour at RT. HNPP/FastRed detection was then performed as described above. Images for quantification were taken with a Leica DM 5000B microscope, while confocal images were taken with a Leica SP5 confocal microscope.

### Immunohistochemistry

For characterization of spinal cord and skin sections, P21 mice were deeply anesthetized with CO_2_ and perfused with 4% PFA in PBS. Intact spinal cords and glabrous skin sections were dissected and post-fixed for 2–4 hours in 4% PFA in PBS at 4 °C, cryoprotected in 30% sucrose in PBS O/N at 4 °C, and embedded in OCT. 20 μM cryosections of spinal cord and skin were washed in PBT (3X 10 minutes), and then blocked in PBS containing 5% lamb serum and 0.3% Triton X-100 for one hour at room temperature. Primary antibodies (ImmunoStar 24112 rabbit anti-CGRP, 1:1000 and Aves GFP-1020 chicken anti-GFP, 1:2000) were diluted in the same buffer and incubated O/N at 4 °C, then washed in PBT (3X 10 minutes). Secondary antibodies (Invitrogen A11039 Alexa 488 conjugated goat anti-chicken and Invitrogen A11012 Alexa 594 conjugated goat anti-rabbit) were incubated in blocking buffer at 1:1000 dilution for one hour at room temperature. Slides were then washed in PBT (3X 10 minutes) and mounted with Fluormount. Confocal images were taken with a Leica SP5 confocal microscope.

For whole mount skin staining, glabrous skin was removed from the hindpaws of P21 mice. Skin sections were then post-fixed for 2 hours in 4% PFA in PBS at 4 °C, rinsed in PBS (3X), and washed with PBT (PBS, 0.5% Triton X-100) O/N at 4 °C. Primary antibodies (same as above) were diluted in blocking solution (75% PBT, 20% DMSO, 5% heat inactivated goat serum), applied to skin sections, and incubated for 72 hours at room temperature. Sections were then washed with PBT (8–10 × 30 minutes), and incubated with secondary antibodies (same as above) diluted in blocking solution for 48 hours at room temperature. After 3X PBT rinses, sections were washed with PBT (8–10 × 30 minutes), and then dehydrated in serial (50, 80, 100%) MeOH/PBS dilutions, with hours per dilution, before dehydration in 100% MeOH O/N at room temperature. Sections were then cleared in 1:1 MeOH and BABB (1 part benzyl alcohol, 2 parts benzyl benzoate) for 1–3 hours at room temperature before being transferred to 100% BABB. After clearing, skin was mounted with BABB and vacuum grease. Confocal images were taken with a Leica SP5 confocal microscope.

### RT-PCR

Adult (>6 weeks old) mice were deeply anesthetized with CO_2_ and perfused with sterile ice-cold PBS. DRGs, spinal cord, and brain were dissected out under RNase free conditions and rapidly frozen on dry ice. Tissue was mechanically homogenized and RNA was isolated using the GeneJet RNA Purification Kit (Fermentas K0731), and cDNA was synthesized with oligo-dT primers using the SuperScript First-Strand Synthesis system (Invitrogen 18080051). RT-PCR was performed on cDNA with primers for *TrpC3* (forward primer CCTGGCTTTCATGATTGGCATGTTC for exon 6, forward primer GAGATCGAGGATGACAGTGATG for junction of exons 7–8, forward primer CGGTATGTTTTGAAAGCACAAGTAGAC for exon 10, reverse primer CAGTTCACCTTCATTCACCTCATC for junction of exons 10–11, reverse primer CACTCACATCTCAGCACACTGGGG for exon 11).

### Plasmid construction and colony PCR

The sequence of transcript spanning exons 6–11 in *TrpC3* KO mice was amplified with PCR using primers listed below. The amplified DNA was purified using the GeneJet PCR purification kit (Fermentas K0702) and ligated using the pGEM-T Easy Vector System (Promega A1360). Ligated plasmids were transformed into DH5-α competent cells, and grown overnight at 37 °C on agarose plates supplemented with 100 μg/mL carbenicillin. Individual colonies were then selected for colony PCR, which was performed with primers for *TrpC3* (forward primer CCTGGCTTTCATGATTGGCATGTTC for exon 6, reverse primer GTGTTGGCTGATTGAGAATGCTG for exon 9, reverse primer CACTCACATCTCAGCACACTGGGG for exon 11). PCR products were sequenced by the Penn genomic sequencing center.

### Western blotting

Adult mice were deeply anesthetized with CO_2_ and DRGs were dissected out and rapidly frozen on dry ice. DRGs were then lysed in lysis buffer (150 mM NaCl, 1% Triton X-100, 0.5% sodium deoxycholate, 0.1% SDS, 50 mM Tris pH 8.0 in ddH_2_0) with protease inhibitor added, for 10 minutes on wet ice. The cells were then centrifuged for 5 minutes at 14,000 x g at 4 °C, and the supernatant was transferred to a new tube and mixed with 2X Laemmli buffer (0.125 M Tris pH 6.8, 20% glycerol, 4% SDS, 0.16% bromophenol blue, 10% 2-mercaptoethanol in ddH_2_0), followed by heating at 95 °C for five minutes. Samples were cooled on ice before being loaded into 4–15% gradient mini-Protean TGX gels (BioRad 456–1084) with an equal volume of additional 2X Laemmli buffer. Gels were run at 150 V in running buffer (25 mM Tris base, 190 mM glycine, 0.1% SDS, pH 8.3 in ddH_2_0) and then transferred to nitrocellulose membrane in transfer buffer (25 mM Tris base, 190 mM glycine, 0.1% SDS, 20% methanol, pH 8.3 in ddH_2_0) for one hour. The membrane was then rinsed 3X in TBST (TBS, 0.1% Tween 20) and blocked in blocking buffer (TBST, 5% milk) for one hour at room temperature. Primary antibody (rabbit anti-TRPC3, a gift from Dr. Craig Montell, UC Santa Barbara, 1:1000 dilution) in blocking buffer was then applied to the membranes, and the membrane was incubated at 4 °C O/N. Following 3 × 10 min washes with TBST, membranes were incubated in goat anti-rabbit-AP antibody (Applied Biosystems T2191, 1:5000 dilution) in blocking buffer for one hour at room temperature. The membranes were then washed again with TBST 3 x 10 min, and AP signal was subsequently detected with CDP-Star (Applied Biosystems T2218) and imaged with a Chemi-Doc system (BioRad).

### DRG dissociation

DRG dissociation was performed as described previously^35^. In short, DRGs were dissected from 3-4 week old mice and collected in ice cold DMEM/F12 (Sigma D6421) supplemented with 10% FBS (Gibco 1008214710) and 1X penicillin/streptomycin (Gibco 15140122) (DH10 media). Afterwards, dissected DRGs were digested in enzyme solution (4 U/ml dispase II (Gibco 17105041), 342 U/ml collagenase Type I (Gibco 17100017) mixture in 1X Ca^2+^ free, Mg^2+^ free HBSS (Sigma H6648)) for 20 minutes at 37 °C with constant agitation. After digestion, the enzyme solution was aspirated and replaced with fresh DH10 media, and DRGs were gently titrated to free the neurons. Suspensions containing the neurons were then pelleted at 400 g for 4 minutes in a swinging bucket centrifuge and resuspended in DH10 media. Cells were plated onto 8 mm coverslips treated with 0.1 mg/mL poly-l-lysine and 20 μg/mL laminin (BD 354232) into 24-well cell culture plates, and bathed with DRG culture after two hours of incubation at 37 °C and 5% CO_2_. Cells were then cultured O/N at these conditions until usage for calcium imaging.

### Calcium imaging

Cultured DRG neurons from all spinal levels of twelve three-week old mice (6 *TrpC3* ^*−/−*^ null and 6 littermate WT control mice) were rinsed 3X with 37 °C calcium imaging buffer (130 mM NaCl, 3 mM KCl, 2.5 mM CaCl_2_, 0.6 mM MgCl_2_, 10 mM HEPES salt, 10 mM glucose, 1.2 mM NaHCO_3_ in ddH_2_0, pH 7.45) and then loaded with 10 μM Fura-2AM dye (Thermo F1201) and 0.02% Pluronic F-127 (Molecular Probes P3000MP) in calcium imaging buffer for 25 minutes at room temperature. Cells were then rinsed 3X with calcium imaging buffer and incubated in the dark at room temperature before imaging. After washing, cells were imaged at 340 and 380 nm excitation to detect intracellular calcium while challenged with 1 mM β-alanine (Sigma 146064) for 60 seconds, then washed for 180 seconds with calcium imaging buffer, and then challenged with 100 mM KCl. Cells were identified as functional and healthy neurons if calcium activity was seen after depolarization was elicited with KCl; cells that failed to respond to KCl were excluded from data analysis. All Fura-2 ratios were normalized to the baseline using the formula *F*_340_ */F*_380_ = (Ratio)/(Ratio_*t*=*0*_), and a response to a stimulus was considered as positive if the peak ratio of a response to a stimulus was >10% above baseline. All images were acquired on a Nikon TI-E inverted microscope and using a Photometric HQ2 CCD camera and Nikon NIS Elements AR software suite. Calcium responses were analyzed afterwards using the NIS Elements software. Figures were drawn using Microsoft Excel and GraphPad Prism. Statistical significances were calculated using GraphPad Prism. Graphs show mean plus standard error.

### Mouse behavior

All animals used for behavior were adult (>6 weeks old) mice housed with a 12-hour light-dark cycle with free access to food and water. For the hot and cold plate tests, mice were placed on the metal surface of an IITC Hot Cold Plate Analgesia Meter, and the temperature of the plate was gradually increased or decreased by 6 °C/min until the mice showed clear signs of discomfort (paw licking, biting, jumping). The temperature of the apparatus at this time was recorded as the pain threshold. Each animal performed 3 trials with an intertrial interval of at least 30 minutes, and the thermal threshold was averaged over all three trials. To measure radiant heat pain, the Hargreaves’ test was used. Mice were placed in a rectangular chamber on the glass surface of the Hargreaves’ apparatus and the radiant heat beam was applied to the hindpaw. The latency for the animal to withdraw from the heat source was measured, and beam intensity was adjusted so that wild type mice displayed a latency to withdraw within 8–12 s. Each paw of the animal was stimulated for 10 trials, with an intertrial interval of at least 10 minutes, and the latency to withdrawal was averaged over all 20 trials.

To measure motor activity, the rotarod test was used. Mice were first trained on the rotarod for a 120 second training session at a constant speed of 4 rpm. Mice were then tested a day later on the rotarod while accelerating the rod at an increase of 6 rpm/min. Each animal performed 3 trials with an intertrial interval of at least 15 minutes, and latency to fall was averaged over all three trials.

To determine sensitivity to mechanical force, the von Frey test was used. Mice were placed in a rectangular chamber on an elevated mesh grid and the plantar surface was stimulated with a von Frey filament, which would bend upon reaching the surface of the hindpaw. We used a 0.8 g filament, which was previously calibrated to induce a response 50% of the time in wild type mice. Each paw of the animal was stimulated for 10 trials, with an intertrial interval of at least 10 minutes, and the latency to withdrawal was averaged over all 20 trials. Sensitivity to dynamic light touch was determined using a brush test. Mice were placed in a rectangular chamber on an elevated mesh grid and the plantar surface of the hindpaw was lightly stimulated with a paintbrush by stroking once across the paw from heel to toe. Each paw of the animal was stimulated for 10 trials, with an intertrial interval of at least 10 minutes, and the latency to withdrawal was averaged over all 20 trials. To determine sensitivity to noxious mechanical force, the pinprick test was used. Mice were placed in a rectangular chamber on an elevated mesh grid and the plantar surface of the hindpaw was stimulated with an Austerlitz insect pin. The pin was gently applied to the plantar surface of the hindpaw without penetrating the skin. Each paw of the animal was stimulated for 10 trials, with an intertrial interval of at least 10 minutes, and the latency to withdrawal was averaged over all 20 trials. Sensitivity to light touch was determined using the sticky tape assay. Mice were placed in an empty cage and habituated for 10 minutes. A square sticker with a quarter inch length was applied to the plantar surface of the hindpaw and the latency to response (grooming, paw shaking, attempt to remove the sticker) was recorded. Each animal performed 3 trials with an intertrial interval of at least 5 minutes, and latency to response was averaged over all three trials.

Itch behavior was conducted as previously described^16^. Briefly, 50 mM β-alanine was injected intradermally into either the back or cheek of the mouse. Mice were first acclimated to the testing chamber for at least 20 minutes, then injected with 50 mM β-alanine or saline into the cheek (10 μL) or back (50 μL). Behavior was then observed for 20 minutes, during which the number of scratching bouts towards the area of injection was quantified. Subsequent data analysis and statistics (student’s t-test) were performed in GraphPad Prism. Graphs show mean plus standard error.

### Data availability

The data generated and analyzed in this study are available from the corresponding authors on reasonable request.
